# Genetic Basis of Ammonium Toxicity Resistance in a Sake Strain of Yeast: A Mendelian Case

**DOI:** 10.1534/g3.113.005884

**Published:** 2013-04-01

**Authors:** Cyrielle Reisser, Cynthia Dick, Leonid Kruglyak, David Botstein, Joseph Schacherer, David C. Hess

**Affiliations:** * Department of Genetics, Genomics and Microbiology, University of Strasbourg, CNRS, UMR7156, Strasbourg, 67083 France; † Department of Biology, Santa Clara University, Santa Clara, California 95053; ‡ Lewis-Sigler Institute for Integrative Genomics and Department of Ecology and Evolutionary Biology; § Howard Hughes Medical Institute; ** Lewis-Sigler Institute for Integrative Genomics and Department of Molecular Biology, Princeton University, Princeton, New Jersey 08544

## Abstract

High concentrations of ammonium at physiological concentrations of potassium are toxic for the standard laboratory strain of *Saccharomyces cerevisiae*. In the original description of this metabolic phenotype, we focused on the standard laboratory strains of *Saccharomyces*. In this study, we screened a large collection of *S. cerevisiae* natural isolates and identified one strain that is resistant to high concentrations of ammonium. This strain, K12, was isolated in sake breweries. When the K12 strain was crossed to the standard laboratory strain (FY4), the resulting tetrads displayed 2:2 segregation of the resistance phenotype, suggesting a single gene trait. Using a bulk segregant analysis strategy, we mapped this trait to a 150-kb region on chromosome X containing the *TRK1* gene. This gene encodes a transporter required for high-affinity potassium transport in *S. cerevisiae*. Data from reciprocal hemizygosity experiments with *TRK1* deletion strains in K12 and BY backgrounds, as well as analysis of the deletion of this gene in the K12 strain, demonstrate that the K12 allele of *TRK1* is responsible for ammonium toxicity resistance. Furthermore, we determined the minimal amount of potassium required for both the K12 and laboratory strain needed for growth. These results demonstrate that the gene encoded by the K12 allele of *TRK1* has a greater affinity for potassium than the standard allele of *TRK1* found in *Saccharomyces* strains. We hypothesize that this greater-affinity allele of the potassium transporter reduces the flux of ammonium into the yeast cells under conditions of ammonium toxicity. These findings further refine our understanding of ammonium toxicity in yeast and provide an example of using natural variation to understand cellular processes.

Single-celled organisms must deal with drastic environmental changes that result in several challenges to internal homeostasis. These external factors include nutrient starvation, temperature changes, osmolarity differences, pH differences, exposure to toxins, and changes in ionic concentrations. The toxicity of ammonium is well established in plant and animal systems (von Wiren and Merrick 2004), but it was not until recently that ammonium toxicity was discovered in *S. cerevisiae* (Hess *et al.* 2006). One aspect that distinguishes ammonium toxicity from other stresses in yeast is that the transcriptional response to the stress is very limited and does not include the environmental stress response signature (Will *et al.* 2010). Furthermore, ammonium toxicity in *Saccharomyces* is proposed to involve the clash between the need for potassium uptake by the cells and the need to exclude excess ammonium ions. Yeast cells can detoxify excess ammonium by fixing the ammonium to carbon skeletons in the form of amino acids and excreting the amino acids into the extracellular space, similar to how mammals detoxify ammonium through the excretion of urea (Hess *et al.* 2006). All of the metabolic pathways involved in ammonium toxicity in yeast and how they interact are not fully characterized.

Hess *et al.* (2006) established that ammonium toxicity in yeast occurs at physiological levels of potassium and high ammonium. In this study we proposed that ammonium ions and potassium ions (both positively charged ions with nearly identical atomic radii) can both be transported by potassium transporters. At physiological levels of potassium, the high concentration of potassium transporters in the cell membrane creates a toxic “leak current” of ammonium into the cell. We observed that yeast cells can detoxify this excess ammonium by increasing the production of amino acids and excreting them into the media (Hess *et al.* 2006). However, if the ammonium concentration is sufficiently high under physiological concentrations of potassium, then the media is toxic to yeast. This link between ammonium toxicity and potassium transport is crucial to the findings in this article.

High levels of ammonium are found in decaying organic matter such as the substrates used in ethanol fermentations. Because fermentation methods and substrates vary across the world, we wanted to examine a collection of yeast strains adapted to these different fermentation processes and test for resistance to ammonium toxicity. Natural variation in yeast has been successfully used to study non-ammonium toxicity stress responses (Will *et al.* 2010), differential use of metabolites (Wenger *et al.* 2010), and the differential ability to restart stuck wine fermentations (Marullo *et al.* 2007).

We screened a collection of 63 *S. cerevisiae* strains for resistance to ammonium toxicity. We identified one strain, K12, previously used for sake fermentation, as being highly resistant to ammonium toxicity. Using bulk-segregant analysis, we identified a region of the genome linked to the casual mutation conferring resistance to ammonium toxicity. Sequence analysis of the region revealed one gene, *TRK1*, with multiple nonconservative mutations. *TRK1* encodes a subunit of the potassium ion transporter and based on its function was an excellent candidate for involvement in ammonium toxicity. Deletion of the *TRK1* gene in the K12 strain background creates a strain fully sensitive to ammonium toxicity. Furthermore, the K12 allele of *TRK1* displays greater affinity for potassium than the standard laboratory allele. We conclude that the K12-allele of *TRK1* confers resistance to ammonium toxicity and provides validation of the proposed mechanism of ammonium toxicity previously suggested. These results are of further interest because they represent rare cases of a single locus being responsible for a large phenotypic effect (resistance to ammonium toxicity) that evolved naturally.

## Material and Methods

### Strains

The *S. cerevisiae* strains used in this study are listed in Supporting Information, Table S1. FY4 (*MAT***a**) was crossed with K12 (*MATα*) on yeast peptone dextrose agar (YPD) (20 g/L bacto-peptones, 10 g/L yeast extract, 20 g/L glucose, 20 g/L agar) media. Diploid hybrids were sporulated for 2–5 d at 30° on 1% potassium acetate media. A total of 25 tetrads containing the four haploid meiotic products were dissected with a Singer instrument MSM.

### Media, growth conditions, and growth quantification

We adapted the phosphate limited medium from Hess *et al.* (2006) in this study. This base salt–mixed medium contained: 0.1 g/L calcium chloride dihydrate, 0.1 g/L sodium chloride, 0.5 g/L magnesium sulfate heptahydrate, 5 g/L ammonium sulfate, 0.05 g/L potassium chloride, 1 g/L sodium phosphate, and 2 g/L glucose, plus vitamins described previously. A total of 20 g/L of ultrapure agarose was added for the solid medium. Variations from this base medium are noted in the text and figure legends. The base ammonium toxicity medium contained 300 μM potassium and 456 mM ammonium.

### Testing of the limiting concentration of potassium needed for growth

The minimum amount of potassium required to grow was tested at concentrations of 300, 150, 75, or 30 μM KCl. Potassium plates contained 76 mM glutamate as the nitrogen source, 12 g/L ultrapure agarose, and salts, vitamins, and metals as described previously. Ten-fold serial dilutions of K12, FY4, and two K12 *trk1Δ*-null allele strains were made from OD_600_ = 0.8–0.00008. Strains grew at 30° for 4 d before they were photographed.

### Bulk segregant analysis

The ammonium resistant K12 strain was crossed with the FY4 reference strain, and the resulting diploid was sporulated. Segregants were scored for ammonium resistance on plates. In total, 30 sensitive and 30 resistant spores were selected and grown individually in 5 mL of YPD for 12~14 hr at 30°. Samples were pooled by phenotype (resistant and sensitive pools) and genomic DNA was extracted from 30 mL of YPD cultures using QIAGEN Genomic-Tips 100/G and Genomic DNA Buffers as per the manufacturer’s instructions. DNA was digested with DNaseI, labeled, and hybridized to Affymetrix yeast tiling arrays as described in Gresham *et al.* (2006). In summary, the log_2_ ratio of probe intensities (sensitive pool/ resistant pool) was plotted across every chromosome for each informative nucleotide to map the region involved in the resistance. The plots of each chromosome were scanned visually for local peaks in intensity.

### Deletion construction

The disruption of the *TRK1* gene in the K12 strain was performed by homologous recombination. In summary, the *TRK1*::*KanMX* cassette was amplified from the corresponding deletion mutant (Winzeler *et al.* 1999). Approximately 400 bp up and downstream of the deleted *TRK1* gene were amplified with the cassette. The fragment was amplified using the primers TRK1.1 (5′-CATCAGAAATGTACGTAGGCA-3′) and TRK1.2 (5′-CCATTAGCATCACTGATTCCA-3′) and the iProof High-Fidelity DNA Polymerase from MP Biomedicals as described by the manufacturer. The resulting cassette was integrated into K12 by lithium acetate transformation and stable G418-resistant transformants were selected. Correct integration was confirmed by polymerase chain reaction.

### Sequence analysis of the *TRK1* gene of different *S. cerevisiae* isolates

Protein sequences of the *TRK1* gene from various *S. cerevisiae* strains were retrieved from the SGD database and from the BLAST server of the Fay Lab (http://www.moseslab.csb.utoronto.ca/spgp/). Because the sequence of the K12 strain was not available, we fully sequenced its *TRK1* gene. Sequence alignments were performed using CLUSTALW2 (Larkin *et al.* 2007) program with default parameters. The Neighbor-Joining tree was constructed using SplitsTree program (Huson and Bryant 2006).

## Results

### Screening of natural isolates of *S. cerevisiae* for resistance to ammonium toxicity

At physiological concentrations of potassium high concentrations of ammonium are toxic to the several standard laboratory strains of yeast, including FY4. Low potassium and high ammonium conditions often are present in decomposing vegetation. Furthermore, several different types of grains, fruits, and vegetables are used as substrates for fermentation by *S. cerevisiae*. Depending on the specific fermentation process, these substrates are in different stages of decomposition. Thus, we hypothesized that certain fermentation strains have been exposed to the conditions of ammonium toxicity.

**Figure 1:**
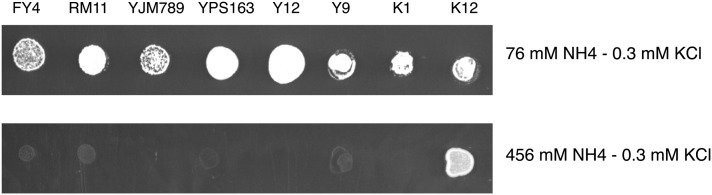
The K12 strain, resistant to high concentration of ammonium. Cells were grown on plate with high (456 mM NH_4_) and low (76 mM NH_4_) concentrations of ammonium with low (300 μM) potassium.

To test this hypothesis, we screened each strain in our collection for resistance to high concentration of ammonium. The collection comprises *S. cerevisiae* strains from various sources, including brewing, baking, wine, laboratory, as well as clinical strains (Schacherer *et al.* 2009) (Table S1). We placed each strain on plates containing low and high concentrations of ammonium (76 mM and 456 mM NH_4_^+^; (Figure 1 and Figure S1). Of the 63 strains tested, we only identified one strain, K12, that was capable of growing in the presence of a high concentration of ammonium. K12 was isolated in sake breweries (Fay and Benavides 2005). Sake strains produce ethanol as well as organic acids and amino acids in the sake mash that contribute to sake aroma and taste (Akao *et al.* 2011).

**Figure 2:**
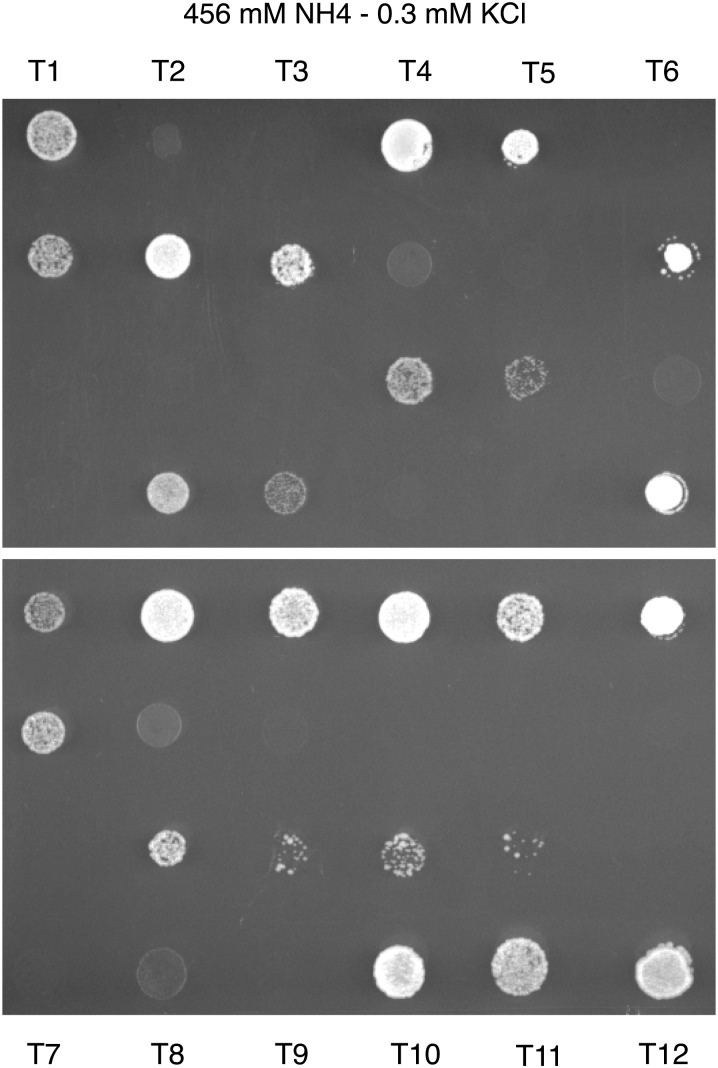
Segregation of ammonium resistance in the cross between K12 and FY4. Segregants were grown on plate with high concentration of ammonium (456 mM NH_4_). All tetrads show a 2:2 segregation of growth and no growth.

To understand the degree of genetic complexity of ammonium resistance, we mated K12, the strain resistant to ammonium toxicity, to the FY4 laboratory strain, which is unable to grow under conditions of ammonium toxicity. Spores from 25 tetrads were tested for their ammonium toxicity phenotypes (Figure 2). Among the four spores from each tetrad, two spores had a growth phenotype and two spores had a no growth phenotype. This classic 2:2 segregation pattern indicates that a single Mendelian locus is responsible for ammonium resistance of the K12 strain.

### Dissection of the genetic origin of the resistance: *TRK1*, a candidate

To determine the genomic location of the gene involved in ammonium resistance, we conducted a bulk segregant analysis (Dunham 2012). In summary, this strategy compares two pools of segregants coming from the K12/FY4 cross: one pool of spores with resistance to ammonium toxicity and a second group spores with sensitivity to ammonium toxicity. The DNA of each pool was genotyped for the thousands of single-nucleotide polymorphisms (SNPs) that differ between them by hybridization on a yeast tiling array. Comparison of these SNP genotypes revealed the region that is involved in the phenotypic variation. For Mendelian locus, these DNA pools should be equal mixtures of the K12 alleles and laboratory alleles across the whole genome except the region near the causal locus or loci. At these loci, the phenotypically “resistant” pool should consist only of the alleles from the K12 strain. We found one such region from the K12/FY4 cross.

**Figure 3:**
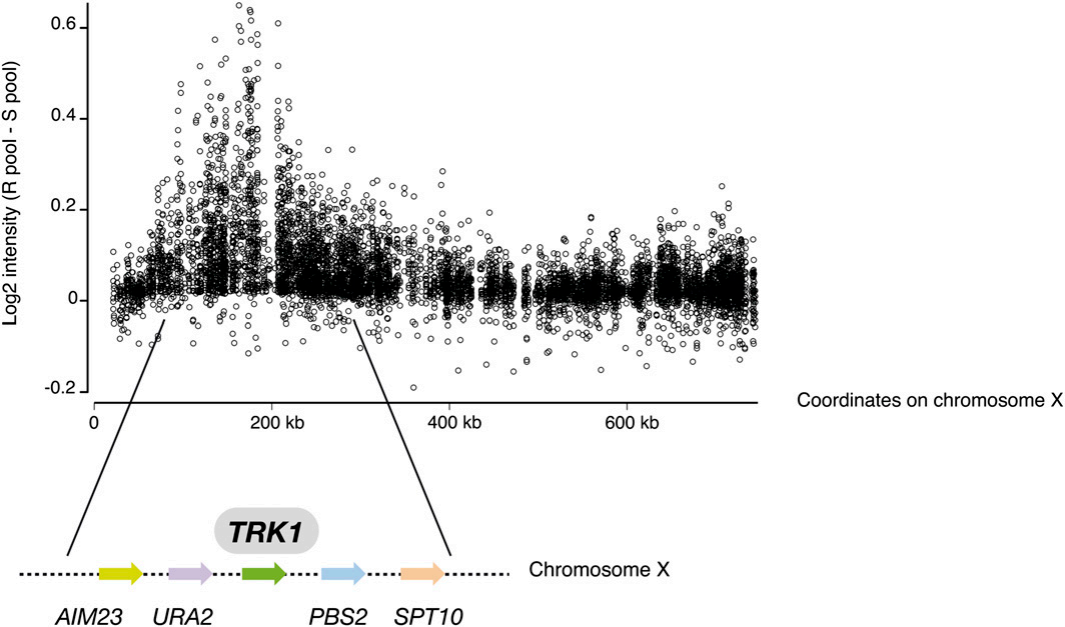
Bulk segregant analysis identifies *TRK1*. Genomic DNAs from pools of sensitive and resistant segregants were hybridized independently to Affymetrix yeast tilling arrays. Plotted here is the log_2_ ratio of probe intensities (sensitive pool/resistant pool) along the chromosome X.

Spores from the K12/FY4 diploid were screened for ammonium resistance and sensitivity. In total, 30 resistant spores were combined in a pool and 30 sensitive spores into another pool. Genomic DNA was extracted from each pool and then hybridized individually on a tiling array (based on the S288c reference genome). Based on previous results, we knew that the genetic divergence between K12 and FY4 is approximately 0.25%, representing a SNP density of 2.3 SNPs/kb (Schacherer *et al.* 2009). Because of this level of polymorphisms, we knew that the regions derived from the K12 strain would hybridize less efficiently to the array. After hybridization, we therefore plotted the log_2_ ratios of probe intensities (sensitive pool/resistant pool; Figure 3). For most of the genome, log_2_ ratios of probe intensities were around 0, showing that the allele frequency of K12 and FY4 is equivalent in both pools. Nevertheless, the bulk segregant analysis identified one linkage peak on chromosome X, which corresponds to less-efficient hybridization to the array of the resistant pool genomic DNA (Figure 3). The mapped region is approximately 150 kb in length and situated between open reading frames YJL171c and YJL095w. It encompasses more than 70 open reading frames. Interestingly, this region contains the *TRK1* gene (YJL139c). This gene is a good candidate for two reasons. First, *TRK1* is located in the middle of the linkage peak (Figure 3). Second, this gene encodes a transporter required for high-affinity potassium transport in *S. cerevisiae* (Gaber *et al.* 1988). Hess *et al.* (2006) hypothesized that ammonium toxicity in yeast occurs via an uncontrolled leak current of ammonium through the potassium channel, making *TRK1* an attractive candidate gene in which one might find allelic differences that cause, by themselves, resistance to ammonium toxicity.

### Reciprocal hemizygosity experiments with the *trk1Δ0* strains

**Figure 4:**
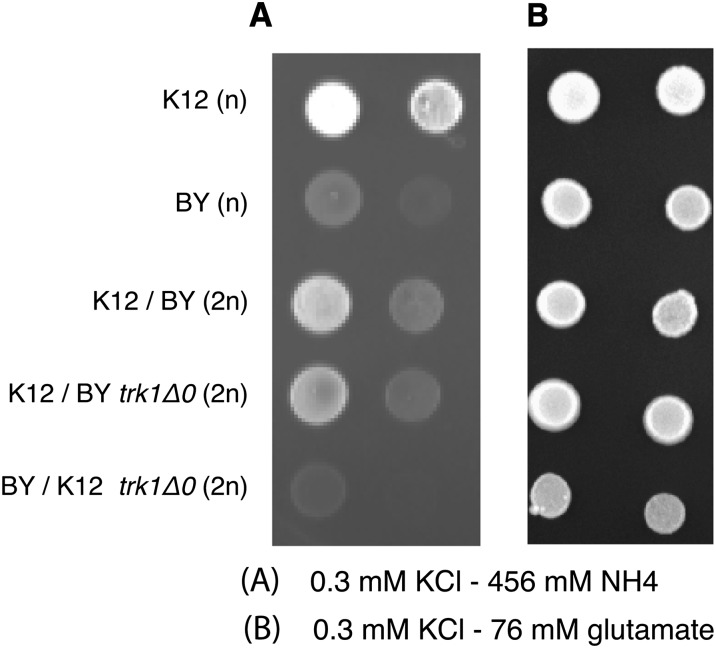
Reciprocal hemizygosity with *trk1Δ0* in K12 and BY backgrounds. Cells were grown on plate with either 456 μM ammonium as a nitrogen source (A) with 300 μM potassium or 76 μM glutamate as a nitrogen (B) source.

To demonstrate involvement of the *TRK1* allele of K12 for the phenotype, we created a deletion strain (*trk1Δ0*) in the K12 and FY4 genetic backgrounds as described in the Methods. Deletion of the high-affinity potassium transporter (*TRK1*) prevented these strains from growing at the low potassium levels used in the ammonium toxicity media (data not shown). These strains were nevertheless analyzed (see the section *Analysis of the trk1Δ0 strains for the ammonium toxicity phenotype* and *Discussion*), but another approach, reciprocal heterozygosity, was taken to test directly the relationship between the K12 *TRK1* allele and resistance to ammonium toxicity. To this end we generated three diploids. The first diploid was made simply by crossing K12 to BY, which we refer to as the control. The second diploid was generated by crossing the *trk1Δ0* in the BY background to K12. This strain only differed from the control in that the only functional allele of *TRK1* was the K12 allele. The third diploid was made by mating the *trk1Δ0* in the K12 background to BY, generating a strain that only differed from the control in that the only functional allele of *TRK1* only was the BY allele. These three diploid strains, as well as the haploid K12 and BY strains, were spotted on both ammonium toxicity medium and control medium (low potassium with glutamate as a nitrogen source; Figure 4). As previously shown, the K12 haploid strain grows well on ammonium toxicity medium whereas the BY haploid strain grows poorly. The K12/BY diploid strain and the K12/BY *trk1Δ0* diploid strain grow well on the ammonium toxicity medium. This finding shows that the K12 phenotype is dominant and that the BY *TRK1* allele is not required for the resistance phenotype. The important result is that the BY/K12 *trk1Δ0* diploid strain grows poorly on the ammonium toxicity medium, which shows that the presence of K12 *TRK1* allele is sufficient for the ammonium toxicity resistance.

### Analysis of the *trk1Δ0* strains for the ammonium toxicity phenotype

**Figure 5:**
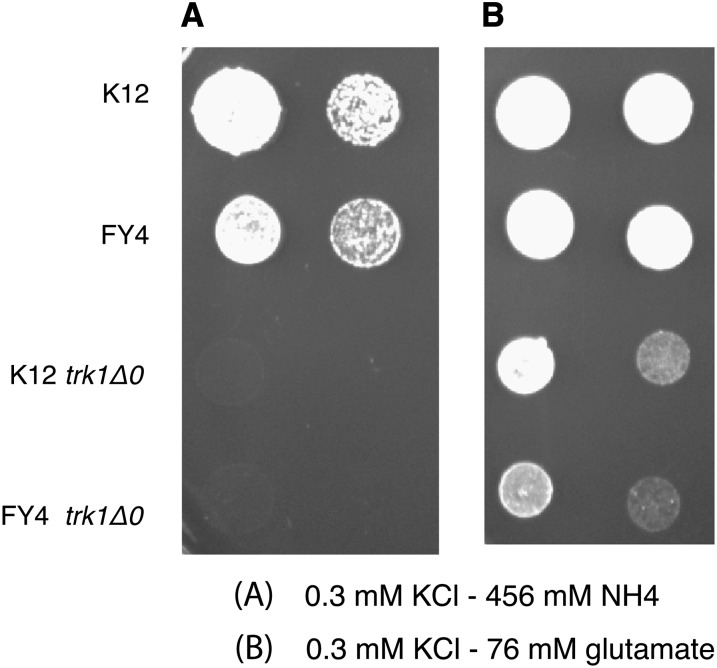
Phenotype of the K12 deletion mutant of *TRK1*. Cells were grown on plate with either 456 μM ammonium as a nitrogen source (A) with 600 μM potassium or 76 μM glutamate as a nitrogen (B) source.

Because TRK1 is the high-affinity transporter for potassium, it was not surprising that *trk1Δ0* deletion strains, regardless of strain background, would not grow on our standard ammonium toxicity media: the potassium concentration was too low (300 μM) for the remaining systems for potassium assimilation. We therefore made plates with 600 μM potassium and either 76 μM glutamate as a nitrogen source or 450 μM ammonium as a nitrogen source. We spotted the K12 and FY4 wild-type strains along with the *trk1Δ0* in both strain backgrounds onto these plates (Figure 5). As expected, the wild-type strains of K12 and FY4 grew well on both plates as the potassium level is too high to induce ammonium toxicity. However, for the *trk1Δ0* in both the K12 and BY backgrounds we observed the ammonium toxicity as predicted by our model of the role of Trk1 in ammonium toxicity (see *Discussion*). Importantly, the *trk1Δ0* strains show equivalent ammonium toxicity in both strain backgrounds—the K12 strain no longer has resistance to ammonium toxicity in the absence of its unique *TRK1* allele (Figure 5). All these data allow us to conclude that *TRK1* is the single locus is responsible for ammonium resistance of the K12 strain.

### Sequence analysis of *TRK1* gene reveals amino acid coding changes unique to the K12 strain

**Figure 6:**
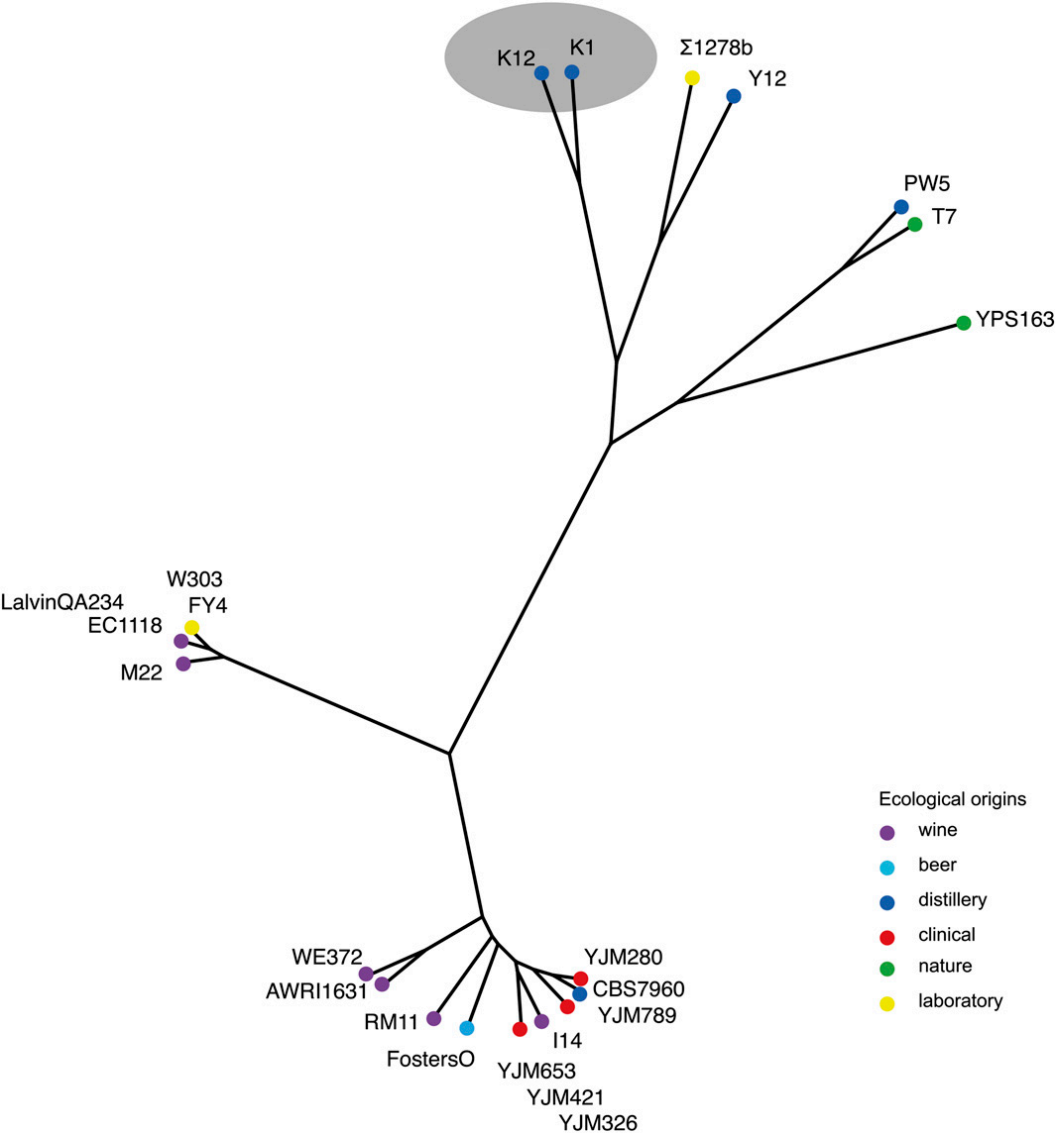
Neighbor-joining tree of the *TRK1* genes of different *S. cerevisiae* isolates.

The relatedness of the *S. cerevisiae* strains was determined based on SNP mapping in all of these strains (Schacherer *et al.* 2009). This study showed the existence of a unique sake cluster that is distinct from wine and laboratory strains. Hence, we explored the genetic diversity of the *TRK1* gene and looked at the phylogeny based on the sequences of this gene. The protein sequences of the *TRK1* gene from 22 various *S. cerevisiae* strains, including K12, were retrieved from the different databases (see *Materials and Methods*). We also sequenced the *TRK1* gene of the K1 strain because K12 and K1 strains are closely related (Schacherer *et al.* 2009). DNA sequence variations were examined, and a neighbor-joining tree was built (Figure 6). As observed at the genome level, the *TRK1* gene follows the same evolution with a close relationship of the strains isolated in sake breweries.

**Figure 7:**
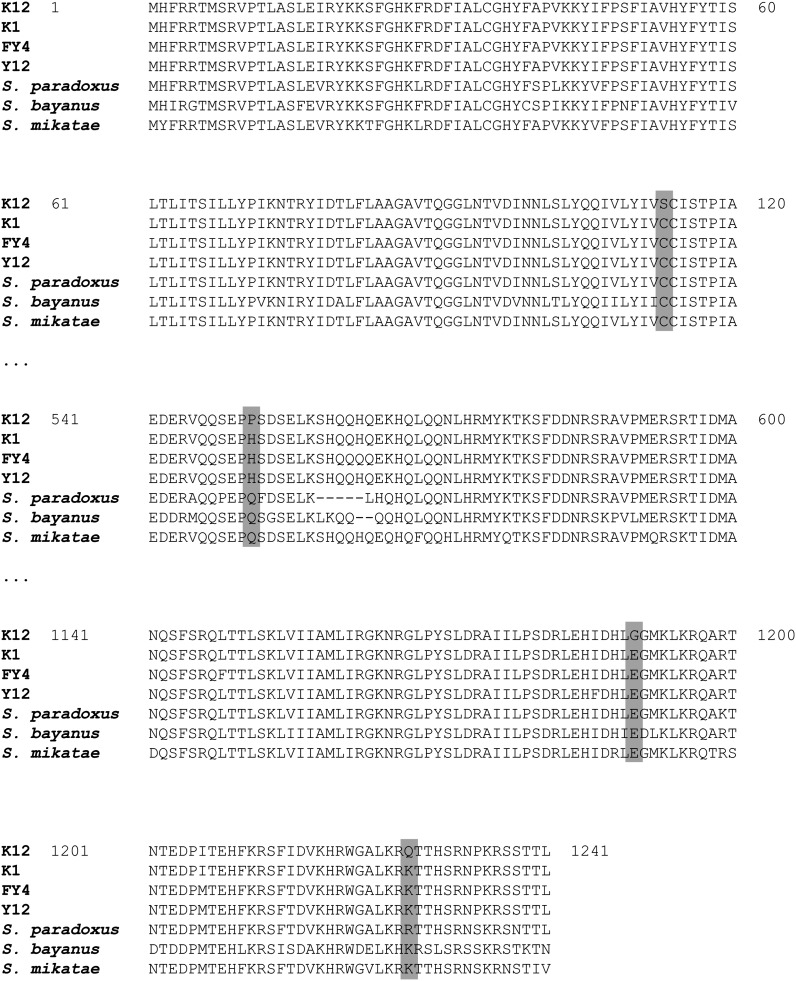
Sequence alignment of *TRK1* genes of *S. cerevisiae* and other *Saccharomyces senso stricto* isolates.

In addition, we compared more closely the protein sequences of K12, K1, Y12, and FY4 strains. Unlike the K12 strain, the K1, Y12, and FY4 strains are sensitive to high concentration of ammonium (Figure S1). Interestingly, comparison between these strains sequences revealed that four amino-acid changes are specific to the K12 strain: 1143 C → S, 551 H → P, 1190 E → G, and 1227 Q → K (Figure 7).

### K12 *TRK1* allele has two amino changes in highly conserved regions of the protein

Two of the changes highlighted in Figure 6, C113S and E1190G, occur in highly conserved regions of the protein based on sequence conservation found in alignments with Trk1 homologs from *S. paradoxus*, *S. bayanus*, and *S. mikatae* (Figure 7). The C113S change occurs in a 104 amino acid stretch of the protein that had 68% identify among these species (including residue 113) and 87.5% strong similarity among these species. The E1190G change occurs in a 118 amino acid stretch of the protein that had 75% identify among these species (including residue 1190) and 92% strong similarity among these species. The E1190G amino acid change is of note because involves a charged amino acid changed to a glycine in the K12 strain. Such a charge change in a conserved region of an ion transporter could have a large effect on protein function.

### K12 *TRK1* allele displays greater affinity for potassium compared with the FY4 allele

**Figure 8:**
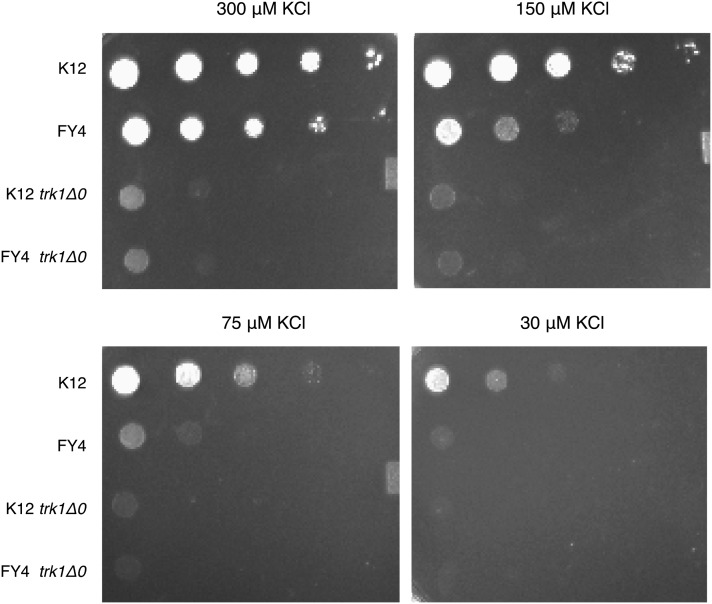
Phenotype of K12 and FY4 strains on limiting potassium media. Cells were grown on plate with different concentrations of potassium (30, 75, 150, and 300 μM KCl).

To test how the amino acid changes in the *TRK1* K12 allele affect protein function, we determined the limiting amount of potassium for growth needed by the K12 and FY4 strains. To accomplish this, we prepared minimal media plates with glutamate as the nitrogen source and four different potassium concentrations ranging from 30 μM to 300 μM. Ten-fold dilutions of K12 and FY4 were plated on these media and grown at 30° for 4 d (Figure 8). As controls, the *trk1Δ0* mutant in both strain backgrounds also was plated to demonstrate how the null allele performs. These results show that FY4 has impaired growth at 150 μM potassium and fails to grow at all at 30 μM potassium. In contrast the K12 strain grows well at 150 μM potassium and only displays impaired growth at 30 μM potassium. The failure of either of the *trk1Δ0* mutants to growth on 150 μM potassium confirms the importance of the *TRK1* gene to growth at limiting concentrations of potassium. Furthermore, these results support the conclusion that the potassium transporter encoded by the K12 allele of *TRK1* has greater affinity for potassium than the potassium transporter encoded by the FY4 allele of *TRK1*.

## Discussion

### Support for the mechanism of ammonium toxicity

A model for ammonium toxicity was proposed that hypothesized ammonium leaking into the cells through potassium channels at physiological concentrations of potassium (Hess *et al.* 2006). The discovery that the K12 strain derives resistance to ammonium toxicity based on a highly divergent allele of the potassium transporter (*TRK1*) supports this link between ammonium and potassium in yeast. Furthermore, the data in this article are consistent with the direct transport of ammonium by the potassium transporter under conditions of ammonium toxicity as predicted (see the section *Model for ammonium toxicity resistance in K12*).

### Model for ammonium toxicity resistance in K12

We speculate that the dramatic alteration in primary amino acid sequence in the K12 Trk1 protein (Figure 7) has the consequence of increasing the affinity for potassium as determined by our potassium limitation experiments. Greater-affinity transporters are often associated with lower flux rates (Epstein and Hagen 1952; Epstein *et al.* 1963) because the transporter holds the cargo for longer during transport due to its greater affinity. If this is the case for the K12 allele of *TRK1*, that allele likely has a lower flux rate for both potassium and ammonium. With a lower ammonium leak rate in K12, these cells would have more time to detoxify the ammonium through excretion of amino acids. This would account nicely for their resistance to ammonium toxicity under these conditions. Of course, direct measurement of the affinity for potassium channels encoded by the different *TRK1* alleles is necessary to validate this model.

### Model for the behavior of the *trk1Δ0* strains

In the absence of the high-affinity, low-flux Trk1 potassium transporter, *S. cerevisiae* must rely on low-affinity, high-flux systems for potassium transport (Gaber *et al.* 1998). As noted previously in this article, low-affinity transporter systems have a greater flux and therefore in the absence of the Trk1 transporter both the potassium and ammonium flux into the cell will be greater. We believe this is why the *trk1Δ0* strains display the ammonium toxicity phenotype at greater concentrations of potassium compared with the wild-type strains. The crucial observation in Figure 4 is that the ammonium toxicity in the *trk1Δ0* strains is identical between the K12 and FY4 strain backgrounds. Thus, the removal of Trk1 from the K12 background removes the resistance to ammonium toxicity. We do not observe any detectable resistance in the K12 background in the absence of Trk1 suggesting that the K12 allele of *TRK1* is the sole determinant of resistance to ammonium toxicity.

## Supplementary Material

Supporting Information
