# Supplementary material for: Genetic Basis of Ammonium Toxicity Resistance in a Sake Strain of Yeast: A Mendelian Case
Source: G3 (Bethesda). 2013 Apr 1;3(4):733–40. doi: 10.1534/g3.113.005884 (PMC3618360; doi:10.1534/g3.113.005884)
Supplement: Supporting Information [file supp_3_4_733__index.html]

Genetic Basis of Ammonium Toxicity Resistance in a Sake Strain of Yeast: A Mendelian Case — Supporting Information 

# Genetic Basis of Ammonium Toxicity Resistance in a Sake Strain of Yeast: A Mendelian Case

## Supporting Information for Reisser *et al.*, 2013

**Files in this Data Supplement:**

- Supporting Information - Figure S1 and Table S1 (PDF, 1 MB)
- Figure S1 - Screening of 63 *S. cerevisiae* on high concentration of ammonium (PDF, 1 MB)
- Table S1 - Description of *S. cerevisiae* strains studied (PDF, 125 KB)
