# Supplementary material for: Genetic Basis of Ammonium Toxicity Resistance in a Sake Strain of Yeast: A Mendelian Case
Source: G3 (Bethesda). 2013 Apr 1;3(4):733–40. doi: 10.1534/g3.113.005884 (PMC3618360; doi:10.1534/g3.113.005884)
Supplement: Supporting Information [file supp_g3.113.005884_005884SI.pdf]

G3 · Genes | Genomes | Genetics

Genetic basis of ammonium toxicity resistance in a sake strain of yeast: a Mendelian case

Reisser C.[1,#], Dick C.[2,#], Kruglyak L.[3,4], Botstein D.[5], Schacherer J.[1, ‡] and Hess DC.[2, ‡]

1. Department of Genetics, Genomics and Microbiology, University of Strasbourg, CNRS, UMR7156, Strasbourg, France.

2. Department of Biology, Santa Clara University, Santa Clara, California, United States of America.

3. Lewis-Sigler Institute for Integrative Genomics and Department of Ecology and Evolutionary Biology, Princeton University, Princeton, New Jersey, United States of America.

4. Howard Hughes Medical Institute, Princeton University, Princeton, NJ, USA

5. Lewis-Sigler Institute for Integrative Genomic and Department of Molecular Biology, Princeton University, Princeton, New Jersey, United States of America.

# These authors contributed equally to this work
‡ These authors contributed equally to this work

Correspondence should be addressed to DH or JS
E-mail: hess.scu@gmail.com, schacherer@unistra.fr

DOI 10.1534/g3.113.005884

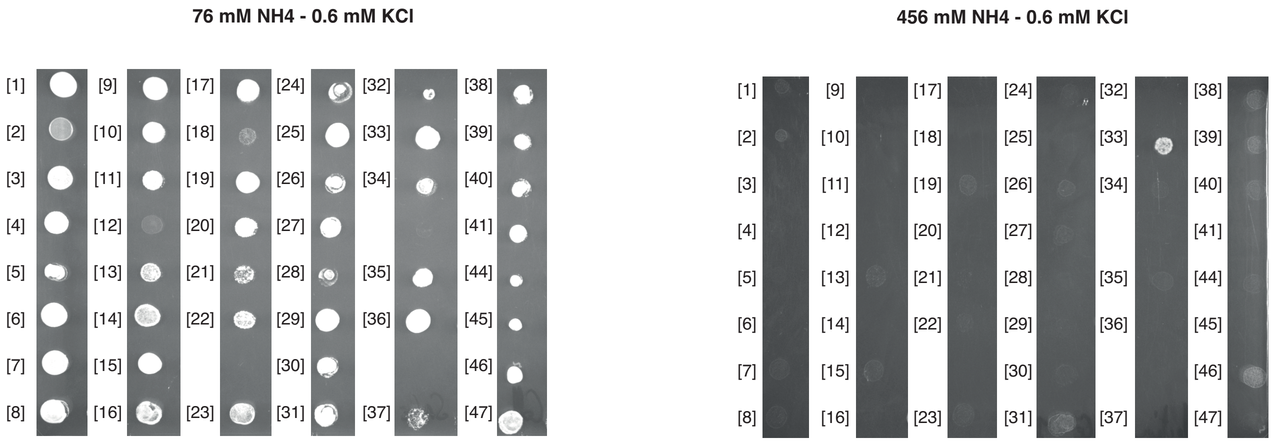

Strains

[1] CBS2888
[2] CBS3093
[3] CBS403
[4] CBS7960
[5] CECT10109
[6] CECT10266
[7] CLIB154
[8] CLIB157
[9] CLIB192
[10] CLIB208
[11] CLIB215
[12] CLIB219
[13] CLIB272
[14] CLIB274
[15] CLIB294
[16] CLIB318
[17] CLIB324
[18] CLIB326
[19] CLIB382
[20] CLIB413
[21] DBVPG1373
[22] DBVPG1399
[23] DBVPG1788
[24] DBVPG1794
[25] DBVPG1853
[26] DBVPG3591
[27] DBVPG4651
[28] DBVPG6041
[29] DBVPG6861
[30] EM93
[31] M22
[32] K1
[33] K12
[34] RM11
[35] T73
[36] TL229
[37] UC1
[38] UC8
[39] WE372
[40] Y10
[41] Y12
[42] Y4
[43] Y5
[44] Y6
[45] Y8
[46] Y9
[47] FY4

Figure S1 Screening of 63 *S. cerevisiae* on high concentration of ammonium. Cells were grown on plate with high (456 mM NH_4) and low (76 mM NH_4) concentration of ammonium.

Table S1: Description of *S. cereviasiae* strains studied

Strains	Sources	Location
CBS2888	Soil South	Africa
CBS3093	Olive-mill wastes	Spain
CBS403	Ginger beer	West Africa
CBS7960	Cane-sugar syrup	Brazil
CECT10109	Prickly pear	Spain
CECT10266	Tanning liquor	Spain
CLIB154	Wine	Russia
CLIB157	Wine	Spain
CLIB192	Baker	France
CLIB208	Baker	China
CLIB215	Baker	New Zealand
CLIB219	Wine	Russia
CLIB272	Beer	Unites States
CLIB274	Baker	Czech republic
CLIB294	Distillery	France
CLIB318	Baker	Holland
CLIB324	Baker	Vietnam
CLIB326	Baker	Australia
CLIB382	Beer	Japan
CLIB413	Fermentation	China
DBVPG1373	Soil	Netherlands
DBVPG1399	Grape	Netherlands
DBVPG1788	Soil	Finland
DBVPG1794	Soil	Finland
DBVPG1853	White Tecc	Ethiopia
DBVPG3591	Cocoa beans	Unknown
DBVPG4651	Tuber Magnatum	Italy
DBVPG6041	Faeces of Man	Unknown
DBVPG6861	Polluted stream water	Brazil
EM93	Rotting Fig	California
M22	Wine	Italy
K1	Sake	Japan
K12	Sake	Japan
RM11	Wine	California
T73	Wine	Spain
TL229	Cheese	France
UC1	Wine	France
UC8	Wine	South Africa
WE372	Wine	South Africa
Y10	Fermentation	Philippines
Y12	Palm Wine	Ivory Coast
Y4	Fruit	Indonesia
Y5	Bili wine	West Africa
Y6	Unknown	French Guiana
Y8	Vineyard	Turkey
Y9	Ragi	Indonesia
