# Supplementary material for: Genetic Basis of Ammonium Toxicity Resistance in a Sake Strain of Yeast: A Mendelian Case
Source: G3 (Bethesda). 2013 Apr 1;3(4):733–40. doi: 10.1534/g3.113.005884 (PMC3618360; doi:10.1534/g3.113.005884)
Supplement: Supporting Information [file supp_g3.113.005884_FigureS1.pdf]

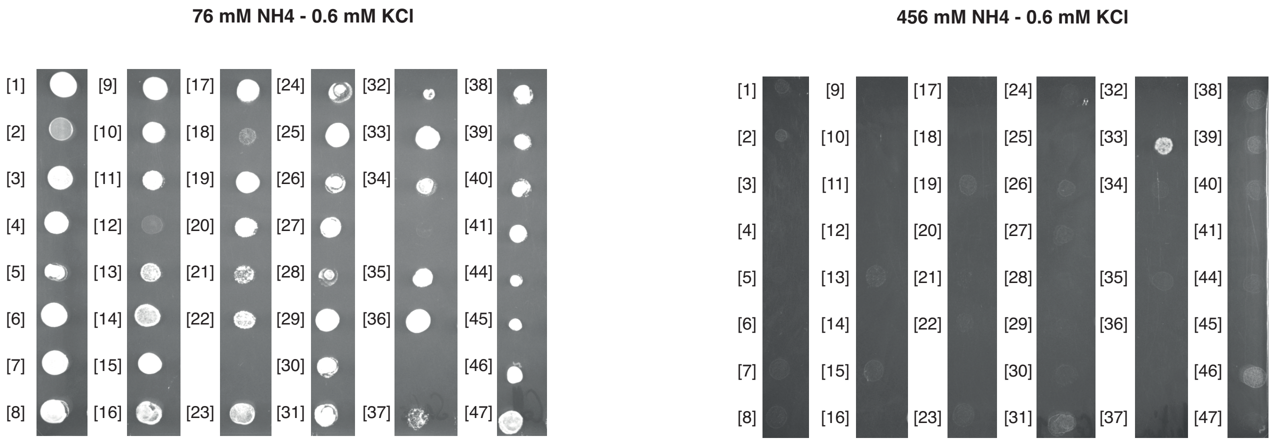

Strains

[1] CBS2888	[6] CECT10266	[11] CLIB215	[16] CLIB318	[21] DBVPG1373	[26] DBVPG3591	[31] M22	[36] TL229	[41] Y12	[46] Y9
[2] CBS3093	[7] CLIB154	[12] CLIB219	[17] CLIB324	[22] DBVPG1399	[27] DBVPG4651	[32] K1	[37] UC1	[42] Y4	[47] FY4
[3] CBS403	[8] CLIB157	[13] CLIB272	[18] CLIB326	[23] DBVPG1788	[28] DBVPG6041	[33] K12	[38] UC8	[43] Y5	
[4] CBS7960	[9] CLIB192	[14] CLIB274	[19] CLIB382	[24] DBVPG1794	[29] DBVPG6861	[34] RM11	[39] WE372	[44] Y6	
[5] CECT10109	[10] CLIB208	[15] CLIB294	[20] CLIB413	[25] DBVPG1853	[30] EM93	[35] T73	[40] Y10	[45] Y8	

Figure S1 Screening of 63 *S. cerevisiae* on high concentration of ammonium. Cells were grown on plate with high (456 mM NH_4) and low (76 mM NH_4) concentration of ammonium.
